# Clinical correlates of chronic cerebrospinal venous insufficiency in multiple sclerosis

**DOI:** 10.1186/1471-2377-12-26

**Published:** 2012-05-15

**Authors:** Bianca Weinstock-Guttman, Murali Ramanathan, Karen Marr, David Hojnacki, Ralph HB Benedict, Charity Morgan, Eluen Ann Yeh, Ellen Carl, Cheryl Kennedy, Justine Reuther, Christina Brooks, Kristin Hunt, Makki Elfadil, Michelle Andrews, Robert Zivadinov

**Affiliations:** 1 Department of Neurology, State University of New York, Buffalo, NY, USA; 2 Department of Biostatistics, University of Alabama, Birmingham, AL, USA; 3 Department of Pharmaceutical Sciences, State University of New York, Buffalo, NY, USA; 4 Buffalo Neuroimaging Analysis Center, Department of Neurology, State University of New York, Buffalo, NY, USA; 5 Department of Neurology, State University of New York, Buffalo, E2 Neurology, Buffalo General Hospital, 100 High Street, Buffalo, NY, 14203, USA

**Keywords:** Multiple sclerosis, Disease progression, Disability, Echo-color Doppler, Venous anomalies, CCSVI

## Abstract

**Background:**

Chronic cerebrospinal venous insufficiency (CCSVI) is a vascular condition characterized by anomalies of the primary veins outside the skull that has been reported to be associated with MS. In the blinded Combined Transcranial (TCD) and Extracranial Venous Doppler Evaluation (CTEVD) study, we found that prevalence of CCSVI was significantly higher in multiple sclerosis (MS) vs. healthy controls (HC) (56.1% vs. 22.7%, *p* < 0.001).

The objective was to evaluate the clinical correlates of venous anomalies indicative of CCSVI in patients with MS.

**Methods:**

The original study enrolled 499 subjects; 163 HC, 289 MS, 21 CIS and 26 subjects with other neurological disorders who underwent a clinical examination and a combined Doppler and TCD scan of the head and neck. This analysis was restricted to adult subjects with MS (RR-MS: *n* = 181, SP-MS: *n* = 80 and PP-MS: *n* = 12). Disability status was evaluated by using the Kurtzke Expanded Disability Status Scale (EDSS) and MS severity scale (MSSS).

**Results:**

Disability was not associated with the presence (≥2 venous hemodynamic criteria) or the severity of CCSVI, as measured with venous hemodynamic insufficiency severity score (VHISS). However, the severity of CCSVI was associated with the increased brainstem functional EDSS sub-score (*p* = 0.002). In logistic regression analysis, progressive MS (SP-MS or PP-MS) vs. non-progressive status (including RR-MS) was associated with CCSVI diagnosis (*p* = 0.004, OR = 2.34, CI = 1.3–4.2).

**Conclusions:**

The presence and severity of CCVSI in multiple sclerosis correlate with disease status but has no or very limited association with clinical disability.

## Background

Multiple sclerosis (MS) is considered a chronic disease of the brain and spinal cord characterized by inflammatory-demyelinating and neurodegenerative features that coexist from early stages [1,2]. The heterogeneous clinical presentation and dissimilar long-term outcomes support a complex MS pathobiology. The recent findings of Zamboni et al. [3-5], who identified a strong association between MS and a condition defined as chronic cerebrospinal venous insufficiency (CCSVI) provide a different viewpoint on the possible cause and progression of MS. However, the relationship of CCSVI to MS pathogenesis is unproven, and sensitivity/specificity findings have ranged from perfect [3] to intermediate [6-13] to null association [14-19]. These contradictory research findings have fueled a polarizing controversy between practitioners and MS patients [20-23]. Therefore a critical objective independent validation of the existence of CCSVI in MS patients and certainly establishing whether CCSVI is a cause, consequence or mere association with MS becomes imperative.

CCSVI has been described as a vascular condition characterized by anomalies of the main extra-cranial cerebrospinal (CS) venous routes that interfere with normal CS venous outflow. These anomalies have been reported to affect the internal jugular veins (IJV), the vertebral veins (VV) and the azygous vein (AZY), and can be detected using a noninvasive combined venous echo-color ultrasound and transcranial (ECD/TCD) Doppler and confirmed by selective venography [3-5]. It has been hypothesized that CS venous anomalies may cause alterations to blood flow that eventually result in iron deposition, decreased brain parenchyma metabolism, degeneration of neurons and characteristic brain injury patterns found in MS [24,25].

In an independent study [9] using identical EC/TCD method as described by Zamboni, our group showed recently a significant association between MS and presence of CCSVI compared to a healthy control group (*p* < 0.001). The goal of the present additional analysis within the initial large heterogeneous MS cohort was to determine the associations of venous hemodynamic findings according to the Zamboni CCSVI criteria with clinical disease severity and classification of disease status as relapsing or progressive.

## Methods

### Study population

#### Ethics statement

The study was approved by the University at Buffalo Human Subjects Institutional Review Board (HSIRB #NEU2490109A). Written informed consent was obtained from all participants.

#### Study design

This cross-sectional study aims to evaluate the clinical correlates of a large cohort of MS patients in relation to the CCSVI criteria [3]. The study represents a post-hoc analysis of the original Combined Transcranial and Extracranial Venous ECD Evaluation (CTEVD) study which was designed to assess the prevalence of CCSVI in a large cohort of patients with MS, healthy controls and controls with other neurological diseases (OND) using specific ECD criteria [3,9]. This CTEVD phase 1 study enrolled 289 MS and 21 clinically isolated syndrome (CIS) patients [9]. The participants received a clinical examination (BWG and DH, that were blinded to the Doppler findings) and an ECD scan of the head and neck (performed by a technician blinded to the subjects’ diagnosis) [9]. The clinical evaluation included neurological assessment using the Kurtzke Expended Disability Status Score (EDSS) based on its separate functional scores and gait endurance [26]. The EDSS was further converted to the MS Severity Scores (MSSS) scale that was designed as a comparative MS population disability assessment tool, to allow comparisons of relative disease severity at all EDSS levels for a given disease duration, using a single clinical assessment at a single point in time [27]. The ECD outcome measure of interest included the presence of CCSVI, as previously defined [3]. Additional ECD outcomes included the individual Doppler Criteria 1–5 and CCSVI severity, as measured by venous hemodynamic insufficiency severity score (VHISS) [3].

### Echo-color doppler data analysis

Combined transcranial and extracranial ECD allows for non-invasive assessment of venous hemodynamic (VH) parameters indicative of CCSVI [3,4]. Cerebral venous return was examined by using the echo-color Doppler (ECD Esaote-Biosound My Lab 25) equipped with 2.5 and 7.5–10 Mhz transducers (Genoa, Italy), with the subject positioned on a tilt bed at 90° and 0° [3,4].

The specific details of subject length of exam, contraindications and limitations, subject assessment, examination guidelines, annotation documentation, specific Doppler parameters, criteria definitions, description of probes, positioning of the subject, hydration status, techniques used, fulfilment of VH criteria and pathology definitions are provided elsewhere [9].

Each subject was assigned a total criteria VH score that was calculated by counting the number of criteria that the subject fulfilled. A subject was considered CCSVI-positive if ≥ 2 VH criteria were fulfilled. Subjects who were not assessed for some VH criterion, due to technical difficulty, were assumed not to have fulfilled that criterion.

The five VH criteria evaluated are: Criterion 1) reflux in the IJVs and/or in the VVs assessed in both sitting and supine postures, Criterion 2) reflux in the deep cerebral veins (DCVs), Criterion 3) B-mode detection of stenoses in the IJVs in the form of annuli, webs, septa, or malformed valves, Criterion 4) absence of ECD signal in the IJV and/or in the VVs, even after forced deep breaths, and Criterion 5) presence of a negative difference in the cross sectional area (CSA) of the IJV. The severity of CCSVI was measured by VHISS, as previously reported [3]. VHISS is based on the sum of pathologic parameters measured for each of the 5 criteria examined. VHISS is ranging from 0 to 16.

### Data analysis

SPSS (SPSS Inc., Chicago, IL, version 15.0) statistical program was used for all statistical analyses. The subjects with relapsing-remitting (RR) MS were categorized as non-progressive MS whereas subjects with relapsing and relapse-free forms of secondary progressive (SP) and primary-progressive (PP) MS were categorized as progressive MS.

One-way ANOVA followed by post-hoc independent sample *t*-tests were used to test for differences in means of continuous demographic variables such as age, age of onset, and disease duration. The χ^2^ test was used for analysis of count variables for categorical data and the Fisher exact test was used where appropriate.

Logistic regression with the non-progressive vs. progressive MS status as the dependent variable, age as a covariate and gender as a factor was also used to assess the role of CCSVI. Analyses were conducted with main effects models containing CCSVI.

The EDSS values were dichotomized into EDSS ≥ 4 and EDSS < 4.0. The EDSS categories were analyzed as the dependent variable in logistic regression with sex and age and either presence of CCSVI or VHISS as predictors. The MSSS was analyzed as the dependent variable with linear regression with sex and age and either presence of CCSVI or VHISS as predictors. VHISS and number of venous hemodynamic criteria fulfilled were analyzed as dependent variable in Poisson regression with sex as a factor and age as a covariate. The EDSS sub-scores were analyzed with ordinal regression with age as a covariate, sex as a factor and either presence of CCSVI or VHISS as predictors.

In order to avoid too many spurious findings due to multiple comparisons, we do not report anything as statistically significant unless the nominal *p*-value was ≤ 0.01 by using two-tailed tests. A trend was assumed with Type 1 error level of 0.1.

## Results

### Demographic and clinical characteristics

The present analysis was restricted to adult subjects with RR-MS (*n* = 181), SP-MS (*n* = 80) and PP-MS (*n* = 12) disease courses. Healthy controls (*n* = 163), subjects with CIS (*n* = 21), pediatric-onset MS (*n* = 10), NMO (*n* = 6) and OND (*n* = 23) were excluded from the original cohort including 499 subjects [9].

Relapsing (*n* = 19) and non-relapsing SP-MS (*n* = 61) were grouped in to a single SP-MS category and similarly relapsing (*n* = 1) and non-relapsing primary (*n* = 11) progressive MS were grouped together in the PP-MS group. Table 1 shows demographic and clinical characteristics of the enrolled MS groups.

**Table 1:** Demographic and clinical characteristics of the enrolled disease groups

**Demographic variables**	**All MS ** ***n*** **= 273**	**RR-MS ** ***n*** **= 181**	**SP-MS ** ***n*** **= 80**	**PP-MS ** ***n*** **= 12**	***p*-value**
Females: Males (% Female)	208: 65 (76.2%)	138: 43 (76.2%)	63: 17 (78.8%)	7: 5 (58.3%)	0.34
Age, years	47.7 ± 10.7	44.7 ± 10.0	53.3 ± 9.7	54.6 ± 8.0	< 0.001
Disease duration*, years	15.2 ± 10.5	12.1 ± 8.3	21.9 ± 12.0	15.8 ± 9.4	< 0.001
Median EDSS* (IQR)	3.0 (4.0)	2.0 (1.5)	6.0 (1.5)	5.75 (3.13)	< 0.001
MSSS	4.05 ± 2.51	3.08 ± 2.12	5.87 ± 2.11	6.06 ± 2.71	< 0.001
Treatment duration, years	3.8 ± 3.7	3.4 ± 3.5	4.8 ± 4.1	3.4 ± 3.0	0.03

* From symptoms.

Female to male ratio: χ^2^ test; Age, disease duration, MSSS, treatment duration: one-way ANOVA; EDSS: Kruskal-Wallis test.

*MS* multiple sclerosis; *PP* primary progressive, *RR* relapsing-remitting; *SP* econdary progressive; *IQR* inter-quartile range; *EDSS* Expanded Disability Status Scale.

No age, sex or disease duration differences were found in the RR-MS and SP-MS in subjects either with or without the presence of CCSVI (all *p* > 0.05 in *t*-tests for age and disease duration and χ^2^ test for sex differences, data not shown). The PP-MS patients with CCSVI were older (59.3 ± 6.3 years) than those without CCSVI (49.8 ± 6.8 years) but the sex distribution and disease duration were similar.

Of the 273 MS, 238/268 (88.8%) were on disease-modifying therapies; data were unavailable for 5 patients (Table 1). No differences in age at disease onset, disease diagnosis or number of relapses during the preceding year were seen between the CCSVI positive or negative groups (data not shown).

### Disease state and CCSVI

Table 2 shows the prevalence of CCSVI classifications for MS patients by subtype of MS. The highest prevalence was seen in SP-MS, followed by PP-MS and RR-MS. In logistic regression correcting for age and sex, progressive MS (SP-MS or PP-MS) vs. non-progressive status (RR-MS) was associated with CCSVI diagnosis (*p* = 0.004, OR = 2.34, CI = 1.3–4.2).

**Table 2:** CCSVI classification by MS subtype

**CCSVI Variables**	**All MS ** ***n*= 273**	**RR-MS ** ***n*= 181**	**SP-MS ** ***n*= 80**	**PP-MS ** ***n*= 12**	***p*-value**
CCSVI diagnosis	153 (56.0%)	89/181 (49.2%)	58/80 (72.5%)	6/12 (50.0%)	0.002
Criterion 1	127 (46.5%)	76 (42.0%)	45 (56.2%)	6 (50.0%)	0.10
Criterion 2	98 (35.9%)	53 (29.3%)	40 (50.0%)	5 (41.7%)	0.005
Criterion 3	173 (63.4%)	115 (63.5%)	52 (65.0%)	6 (50.0%)	0.60
Criterion 4	28 (10.3%)	12 (6.6%)	15 (18.8%)	1 (8.3%)	0.012
Criterion 5	31 (11.4%)	12 (6.6%)	19 (23.8%)	0 (0%)	< 0.001
Number of abnormal criteria	1.7 ± 1.1	1.5 ± 1.1	2.2 ± 1.1	1.5 ± 1.0	< 0.001
VHISS	2.9 ± 1.9	2.6 ± 1.8	3.7 ± 2.0	2.5 ± 1.6	< 0.001

*MS* multiple sclerosis; *PP* primary progressive, *PR* progressive relapsing; *RR* relapsing-remitting; *SP* secondary progressive; *VHISS* venous hemodynamic insufficiency score.

As expected, VHISS was significantly higher for MS patients diagnosed with CCSVI (Mean ± SD: 4.20 ± 1.4) than for subjects without CCSVI (Mean ± SD: 1.28 ± 1.0; *p* < .001 from a Mann–Whitney test). Table 2 gives the distribution of VHISS for the MS disease groups. The MS disease groups (*p* < 0.001, Kruskall-Wallis) significantly differed in VHISS.

### Disability status and CCSVI diagnosis

The three MS subtypes significantly differed on total EDSS score (*p* < 0.001, Kruskall-Wallis test) and all EDSS sub-scores (*p* < 0.001 for each, Kruskall-Wallis test). The probability of EDSS ≥ 4 was not associated with the presence of CCSVI (*p* = 0.42) in logistic regression correcting for age and sex. In ordinal regression correcting for sex and age, the presence of CCSVI diagnosis exhibited a trend with the EDSS brain stem sub-score (*p* = 0.074).

Table 1 gives the distribution of MSSS for the MS disease groups. No evidence for differences in mean MSSS ± SD for subjects diagnosed with CCSVI (4.17 ± 2.6) compared to subjects without CCSVI (3.88 ± 2.3, *p* = 0.60 from a Mann–Whitney test) were found. Similarly linear regression analyses (correcting for sex and age) did not show association with CCSVI (*p* = 0.49).

### Disability status and CCSVI severity

The VHISS exhibited a trend with increasing age (Slope B ± SE: 0.007 ± 0.0033, Wald χ^2^ = 4.54, *p* = 0.033) in Poisson regression with sex as a factor and age as a covariate. However there was no evidence for associations with EDSS.

VHISS was associated with the brainstem EDSS sub-score (*p* = 0.002) and trends were found for the pyramidal (*p* = 0.026) and cerebellar (*p* = 0.051) sub-scores. The probability of EDSS ≥ 4 was not associated with the severity of CCSVI as assessed by VHISS (*p* = 0.056) in logistic regression correcting for age and sex.

## Discussion and conclusion

The present study, a post hoc analysis of the original CTEVD study, which aimed primarily to determine the prevalence of CCSVI in MS patients, provides supplementary informative data to better understand the relation between MS and CCSVI criteria with a specific emphasis on disease and disability status. The original CTEVD study showed an increased prevalence of CCSVI in MS but substantially lower than the originally reported than the 100% sensitivity and 100% specificity reported by Zamboni *et al*. [3] as only 56.1% of MS patients and 38.1% of CIS patients presented with abnormal venous ECD/TCD [9].

Bastianello et al. in a recent multicenter study evaluating 710 patients and using the Zamboni criteria [3] showed a significant association between presence of CCSVI (identified in 86%) and progressive disease [28]. The positive CCSVI patients had also higher disability and a later disease onset as compared to the MS patients without CCSVI. We can speculate that the presence of CCSVI related to certain structural abnormalities as incompetent valves or abnormal intra-luminal structures as septums, flaps or webs can worsen with aging and/or disease progression [29]. In fact, another study showed that CCSVI was diagnosed in 50% of MS patients but was seen also in 36% of the healthy controls [30]. No correlation between presence of CCSVI and EDSS or progressive disease was found, although this can be related to the small number of progressive patients (15/84) enrolled in this study [30]. Another smaller scale study found no association between disease status and CCSVI [16].

Although our large prevalence study showed higher frequency of CSSVI in progressive MS compared to relapsing MS, we did not obtain evidence for significant associations with disability. The divergence of the prevalence and disability data adds to the known difficulties of understanding and measuring the effects of the two key pathological mechanisms in MS – inflammatory vs. neurodegeneration – by introducing the presence of a VH dysfunction as a new variable. Hemodynamic venous dysfunction may result in secondary tissue hypoperfusion that may be associated with a continuous neurodegenerative process. Although VH dysfunction may be less related to the inflammatory aspects of the disease, we speculate that abnormal VH may progressively deteriorate in the context of the inflammatory milieu and activation of the vascular endothelium that occurs in MS [31]. Our previous findings in a OND group showing a high prevalence of CCSVI in the majority of patients diagnosed with inflammatory diseases (i.e., anti-phospholipid antibody syndrome, thyroiditis) [9]. CCSVI findings, if present at all, may reflect: i) benign anatomical variants ii) slower venous outflow due to global cerebral hypoperfusion or iii) alterations to venous walls secondary to a chronic inflammation milieu.

In conclusion, our results indicate that features of CCSVI have no relationship to disability in MS. Further prospective studies including multimodal analysis (i.e., MRI/MRV, ECD/TCD) may help in providing more light into the role of CCSVI or of VH dysfunction in MS.

## Competing interests

Dr. Weinstock-Guttman received compensation for speaking from Teva Neuroscience, Biogen Idec, EMD Serono and Novartis. She also received financial support for research activities from National Institute of Health, National Multiple Sclerosis Society, National Science Foundation, EMD Serono, Biogen Idec, Teva Neuroscience, Novartis, Cyberonics and the Jog for the Jake Foundation.

Murali Ramanathan received research funding or consulting fees from EMD Serono, Biogen Idec, Allergan, Netezza, Pfizer, Novartis, the National Multiple Sclerosis Society, the Department of Defense, Jog for the Jake Foundation, the National Institutes of Health and National Science Foundation. He received compensation for serving as an Editor from the American Association of Pharmaceutical Scientists. These are unrelated to the research presented in this report.

Ralph Benedict has received compensation for serving on Advisory Panels for Biogen, EMD Serono, Merck, Novartis, and Bayer. He has received CME funding from Bayer, Merck, and Biogen and grant support from NMSS, NIH, Biogen, and Shire.

Dr. Zivadinov has received speaker honoraria and consultant fees from Teva Neurosciences, Biogen Idec, Questcor, Genzyme and EMD Serono; and received research support from the National Multiple Sclerosis Society, the Biogen Idec, Teva Neuroscience, Teva Pharmaceuticals, Genzyme, Questcor, Bracco and Greatbatch.

This study was funded by internal resources of the Buffalo Neuroimaging Analysis Center and Baird MS Center, the Jacobs Neurological Institute, University of Buffalo. In addition, we received support from the Direct MS Foundation, the Jacquemin Family Foundation and from smaller donors.

The results from the CTEVD study led to the organization of the IRB approved, unblinded, open-label descriptive study into CCSVI that included patients with either possible or definite MS. This fee-for-service registry study was designed to enhance utilization of data on venous anomalies obtained on individuals who sought information on CCSVI status. This study is no longer active.

## Authors’ contributions

BW-G contributed to study design, oversaw all clinical aspects of the project including clinical data acquisition, data analysis and interpretation and manuscript preparation. MR contributed to study design, data analysis and interpretation and manuscript preparation. RZ contributed to study design, MRI data acquisition, data interpretation and manuscript preparation. KM contributed to Doppler data acquisition. DH contributed to clinical data acquisition and interpretation. RHBB contributed to study design and interpretation of findings. CM contributed to data analysis. EAY contributed to patient recruitment and clinical data acquisition. EC contributed to data acquisition and analysis. CK, JR, CB, KH and MA contributed to patient recruitment and study coordination. ME contributed to Doppler data acquisition. All authors read and approved the final manuscript.

## Pre-publication history

The pre-publication history for this paper can be accessed here:

http://www.biomedcentral.com/1471-2377/12/26/prepub
